# Sequential Infection of *Aedes aegypti* Mosquitoes with Chikungunya Virus and Zika Virus Enhances Early Zika Virus Transmission

**DOI:** 10.3390/insects9040177

**Published:** 2018-12-01

**Authors:** Tereza Magalhaes, Alexis Robison, Michael C. Young, William C. Black, Brian D. Foy, Gregory D. Ebel, Claudia Rückert

**Affiliations:** Arthropod-borne and Infectious Diseases Laboratory, Department of Microbiology, Immunology, and Pathology, Colorado State University, Fort Collins, CO 80523, USA; lexir5394@gmail.com (A.R.); emceeyoung@gmail.com (M.C.Y.); William.Black@colostate.edu (W.C.B.IV); Brian.Foy@colostate.edu (B.D.F.); Gregory.Ebel@colostate.edu (G.D.E.)

**Keywords:** mosquitoes, arboviruses, coinfection, chikungunya, Zika, sequential infection

## Abstract

In urban settings, chikungunya, Zika, and dengue viruses are transmitted by *Aedes aegypti* mosquitoes. Since these viruses co-circulate in several regions, coinfection in humans and vectors may occur, and human coinfections have been frequently reported. Yet, little is known about the molecular aspects of virus interactions within hosts and how they contribute to arbovirus transmission dynamics. We have previously shown that *Aedes aegypti* exposed to chikungunya and Zika viruses in the same blood meal can become coinfected and transmit both viruses simultaneously. However, mosquitoes may also become coinfected by multiple, sequential feeds on single infected hosts. Therefore, we tested whether sequential infection with chikungunya and Zika viruses impacts mosquito vector competence. We exposed *Ae. aegypti* mosquitoes first to one virus and 7 days later to the other virus and compared infection, dissemination, and transmission rates between sequentially and single infected groups. We found that coinfection rates were high after sequential exposure and that mosquitoes were able to co-transmit both viruses. Surprisingly, chikungunya virus coinfection enhanced Zika virus transmission 7 days after the second blood meal. Our data demonstrate heterologous arbovirus synergism within mosquitoes, by unknown mechanisms, leading to enhancement of transmission under certain conditions.

## 1. Introduction

Chikungunya virus (CHIKV; genus *Alphavirus*) and Zika virus (ZIKV; genus *Flavivirus*) are both arthropod-borne viruses that have recently emerged in the Americas. CHIKV infections were first detected in the Caribbean in late 2013 [1], while ZIKV reached northeast Brazil at roughly the same time but was only detected about one year later [2]. Clinical symptoms of Chikungunya Fever and Zika Fever are highly similar and include fever, arthralgia, myalgia, rash, and conjunctivitis [3]. In addition, both viruses can cause severe disease manifestations such as persistent arthralgia or neurological symptoms [3,4]. ZIKV has also been associated with infection of the developing fetus in pregnant women, resulting in Zika congenital syndrome [5]. In urban settings, both viruses are transmitted by *Aedes aegypti* and *Aedes albopictus* mosquitoes and have rapidly spread across nearly all areas of the Americas where these mosquitoes are established. The main vector, *Ae. aegypti*, is highly anthropophilic. Females generally feed on humans several times throughout their lives, including more than once per gonotrophic cycle [6,7]. The recent global spread of CHIKV and ZIKV provides ample opportunities for coinfections within mosquitoes or people.

With on-going transmission of both viruses throughout the Americas, cases of CHIKV/ZIKV coinfection have been increasingly reported over the last few years [8,9,10,11,12,13,14,15,16]. Despite this, there is still a lack of information regarding the impact of coinfection on transmission potential, epidemiology, and disease severity. Additionally, it remains unknown whether human coinfections are more likely to be the result of an individual mosquito transmitting two viruses with the same bite or the result of two separate mosquitoes transmitting each virus. In 2010, a single CHIKV/DENV coinfected *Ae. albopictus* mosquito was found during an outbreak in Gabon [17], yet most surveillance efforts pool mosquitoes and do not allow for the detection of coinfected individual mosquitoes. While evidence for coinfection in the field is scarce, it has recently been shown that *Ae. aegypti* mosquitoes exposed to both CHIKV and ZIKV in the same blood meal can become infected with and transmit both viruses at the same time [18,19]. It is thus conceivable that humans would be infected with CHIKV and ZIKV by the bite of a single mosquito. However, these studies assumed mosquitoes would feed on a host viremic for both viruses and acquire CHIKV and ZIKV from one individual. Alternatively, mosquitoes may be exposed to one virus first and later feed on a second host infected with another virus. This sequential exposure to the two viruses may differ significantly from simultaneous exposure. Established viral infections may alter mosquito immune responses through activation or suppression of antiviral responses [20], which may reduce or enhance a subsequent virus infection. Also, viruses may compete for resources resulting in reduced virus replication of a competing second virus. Several other viral–viral interactions may result in suppression or enhancement of a second viral infection [21]. Moreover, the extent to which coinfection occurs in natural transmission cycles is surprisingly poorly understood.

The effects of simultaneous versus sequential infections of mosquitoes with arboviruses on virus transmission can differ in vitro and in vivo [22,23,24,25,26]. In whole mosquitoes, Nuckols et al. [26] reported that *Ae. aegypti* and *Ae. albopictus* infected sequentially with CHIKV and DENV were able to transmit both viruses as opposed to when mosquitoes were infected simultaneously [26]. Additionally, Le Coupanec et al. [27] recently found that CHIKV coinfection with DENV can enhance DENV replication in *Ae. aegypti* salivary glands, which may also increase DENV transmission [27]. Another recent study exposed *Ae. aegypti* to CHIKV and ZIKV, or ZIKV and DENV-2 sequentially, and found that two viruses could be detected in head squashes of mosquitoes, but the authors did not include single infected controls for comparison [28]. Importantly, since saliva was not collected and no single infected controls were analyzed, it remains unknown how sequential infection impacted vector competence and virus transmission in this study. 

**Figure 1:**
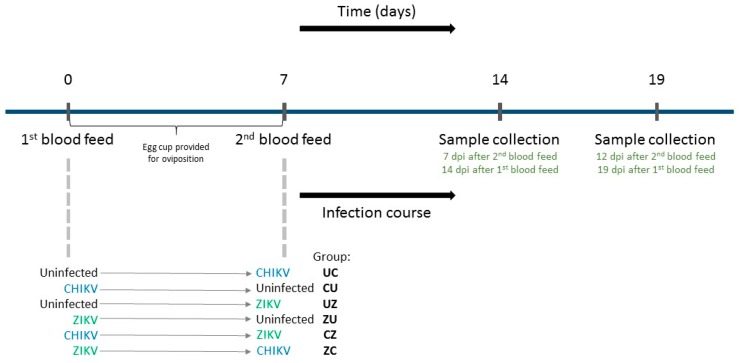
Experimental design of sequential infections of adult *Ae. aegypti* (Poza Rica). A first blood meal with uninfected blood, CHIKV, or ZIKV was given to the mosquitoes, and 7 days later a second blood meal was offered with uninfected blood, CHIKV, or ZIKV. Sample collection (bodies, legs/wings, and saliva) was performed at 7 and 12 days post infection (dpi) following the second blood meal.

We have previously shown that simultaneous exposure of *Ae. aegypti* to both CHIKV and ZIKV slightly reduced ZIKV infection rates compared to single infected controls [19], suggesting that these viruses may interact in mosquitoes in vivo. In the present study, we thus investigated how sequential infection of *Ae. aegypti* mosquitoes with CHIKV and ZIKV would impact vector competence, i.e., the ability of the mosquito to acquire and transmit these viruses. We provided *Ae. aegypti* mosquitoes with two blood meals 7 days apart, each containing either CHIKV, ZIKV, or an uninfected blood meal (Figure 1). We then collected saliva, legs/wings, and bodies 7 and 12 days after the second blood meal to compare infection, dissemination, and transmission rates between mosquitoes exposed to only one virus or both viruses. We performed three replicate experiments and compared single infected to dual infected mosquitoes to fully evaluate the impact of sequential infection on vector competence of both CHIKV and ZIKV. Overall, our study provides evidence that sequential arbovirus infection of *Ae. aegypti* mosquitoes with CHIKV and ZIKV can enhance virus transmission.

## 2. Materials and Methods

### 2.1. Mosquitoes and Viruses

*Ae. aegypti* Poza Rica [29] were maintained under the following standard insectary conditions: 70% relative humidity, 12/12 light/dark cycle, and 28 °C. Mosquitoes were raised to adults and kept with water and a sugar source ad libitum until being used for the experimental blood meals.

ZIKV PRVABC59 (GenBank: KU501215.1; Vero passage 4) and CHIKV 99659 (GenBank: KJ451624.1; Vero passage 2) were propagated in Vero (CCL-81) cells, and frozen stocks were prepared. Frozen stock titers were 3 × 10^7^ PFU/mL and 1.8 × 10^7^ PFU/mL for ZIKV and CHIKV, respectively. 

### 2.2. Sequential Infections with CHIKV and ZIKV, and Sample Collection

Female mosquitoes, 5–7 days old, were sequentially blood-fed with uninfected blood, blood containing ZIKV, or blood containing CHIKV, in different chronological orders (Figure 1). Briefly, a first blood meal was given to the mosquitoes at day 0 and an egg cup was provided to allow for oviposition. Seven days later a second blood meal was offered. The groups, according to the sequential order of the blood meals, were (1) uninfected blood 1st, CHIKV 2nd (group UC); (2) CHIKV 1st, uninfected blood 2nd (group CU); (3) uninfected blood 1st, ZIKV 2nd (group UZ); (4) ZIKV 1st, uninfected blood 2nd (group ZU); (5) CHIKV 1st, ZIKV 2nd (group CZ); and (6) ZIKV 1st, CHIKV 2nd (group ZC). This experiment was replicated three times and the same viral stocks of CHIKV and ZIKV were used for all blood feeds.

The artificial blood meals were carried out with a mix of defibrinated calf blood (Colorado Serum Company) and medium with or without virus. Frozen virus stocks supplemented with 1 µL of a 7.5% sodium bicarbonate solution per 100 µL of virus were used for the blood meals. Virus titers in the final blood meal were approximately 4.5 × 10^6^ PFU/mL for CHIKV and 7.5 × 10^6^ PFU/mL for ZIKV. The blood meal contents were added to glass feeders sealed with hog gut and connected to a 37 °C water bath. Mosquitoes were allowed to feed for approximately 45 min, after which they were anesthetized at 4 °C and sorted. Blood-fed mosquitoes were transferred to clean cartons and given water and sugar *ad libitum*.

The first sample collection was performed 7 days after the second blood meal (7 days post-infection, 7 dpi), and another collection was performed 12 days after the second blood meal (12 dpi). In all cases, anesthetized mosquitoes first had their legs and wings dissected/collected. Next, the mosquito proboscis was inserted in a capillary tube containing immersion oil (salivation stimulant) for 30 min for saliva collection. Lastly, after salivation, whole bodies were collected. Capillaries containing saliva samples were placed in 100 µL of mosquito diluent (1 × PBS containing 20% FBS, 50 µg/mL Penicillin/Streptomycin, 50 µg/mL Gentamycin and 2.5 µg/mL Fungizone), and tubes were then centrifuged at maximum speed for 5 min at 4 °C. Legs/wings and body samples were placed in 200 µL of mosquito diluent in tubes containing sterilized 1/4” stainless steel beads (Glenn Mills); these samples were homogenized for 1 min at 24 Hz using a tissue homogenizer (Retsch Mixer Mill MM400). All samples were kept at −80 °C.

### 2.3. Viral RNA Extraction and Quantitative Real-Time Reverse Transcriptase PCR (qRT-PCR)

Viral RNA was extracted from individual samples using the Mag-Bind Viral DNA/RNA 96 kit (Omega Bio-tek), following the manufacturer’s protocol, and the KingFisher Flex System. RNA samples were kept at −80 °C until further use.

Quantitative real-time reverse-transcriptase PCR (qRT-PCR) was performed with specific primers and probes for CHIKV and ZIKV [19] in a multiplex reaction. RNA standards were generated for both viruses as previously described [19] and included in each qRT-PCR plate.

### 2.4. Statistical Analysis

Logistic regression using GLIMMIX in SAS was used to compare the proportions of infected samples (body, legs/wings, and saliva) from three replicate experiments. We compared groups with the same extrinsic incubation at the time of sampling for each virus—these groups were also fed with the same infectious blood meal (virus aliquot) for the respective virus. For CHIKV infections, we made the following comparisons: UC vs. ZC and CU vs. CZ. For ZIKV infections, we made the following comparisons: UZ vs. CZ and ZU vs. ZC. Replicate information was accounted for in this analysis and significant differences (*p* < 0.05) reported are thus generally differences seen in all three replicates. We also used a Chi-Square test to determine heterogeneity among replicates. Mosquito numbers from individual replicates and results of the Chi-Square test are shown in Supplementary Table S1.

In addition, analysis of variance (ANOVA) with pre-planned contrasts in SAS was used to compare CHIKV and ZIKV genome copies among these same groups. Replicate information was again accounted for in this analysis. For copy number comparisons, we used log_10_(x + 1), to allow inclusion of zeros (negative samples). Since we included zeros, data on genome copies were considered non-normally distributed in the analysis.

## 3. Results

*Ae. aegypti* mosquitoes were first exposed to either CHIKV, ZIKV, or an uninfected blood meal, given the opportunity to lay eggs and again fed with either CHIKV, ZIKV, or an uninfected blood meal 7 days after the initial blood meal, resulting in six treatment groups (Figure 1). Mosquitoes from each group were dissected 7 and 12 days after the second blood meal (7 dpi and 12 dpi), and mosquito bodies, legs/wings, and saliva were tested for viral RNA by qRT-PCR. 

### 3.1. Infection and Dissemination Rates

We first determined the proportion of mosquitoes that became infected with either virus (infection rates) by analyzing mosquito body samples (Table 1, ‘Infection’). Infection rates for CHIKV and ZIKV provided in the first blood meal (groups CU and CZ for CHIKV; groups ZU and ZC for ZIKV) were high (>98% for CHIKV; >86% for ZIKV), and there was no significant difference between groups that were infected with one virus or two (CU vs. CZ and ZU vs. ZC). Once infection is established, mosquitoes usually remain infected, so that infection rates were not significantly different between 7 and 12 dpi. An important question, however, was whether an established infection with one arbovirus would impact infection with a second virus. So, we next determined the infection rates of those groups presented in the second blood meal with CHIKV (UC and ZC) or ZIKV (UZ and CZ). The infection rates were again high for all groups (100% for CHIKV; >96% for ZIKV), and an established infection with CHIKV or ZIKV had no significant impact on ZIKV and CHIKV infection rates (UC vs. ZC; UZ vs. CZ). There was also no significant difference in the infection rates at 7 and 12 dpi. Due to the high infection rates after both the first and second blood feed, the majority of mosquitoes exposed to both CHIKV and ZIKV were co-infected at 7 and 12 dpi independent of the order of exposure (96% of CZ and 89% of ZC).

Once infection has been established, viruses must exit the midgut and disseminate throughout the mosquito to reach the salivary glands. We measured whether mosquitoes have a disseminated infection by screening the legs and wings (legs/wings) for virus. We thus compared the proportions of mosquitoes with virus in legs/wings between the groups as described above for infection rates (Table 1, ‘Dissemination’). Both CHIKV and ZIKV had disseminated to the legs and wings by 7 and 12 dpi in nearly all mosquitoes (>94% for CHIKV; >84% for ZIKV), and we observed no significant differences in dissemination rates between single and dual infections.

### 3.2. CHIKV and ZIKV Load in Bodies and Legs/Wings

**Figure 2:**
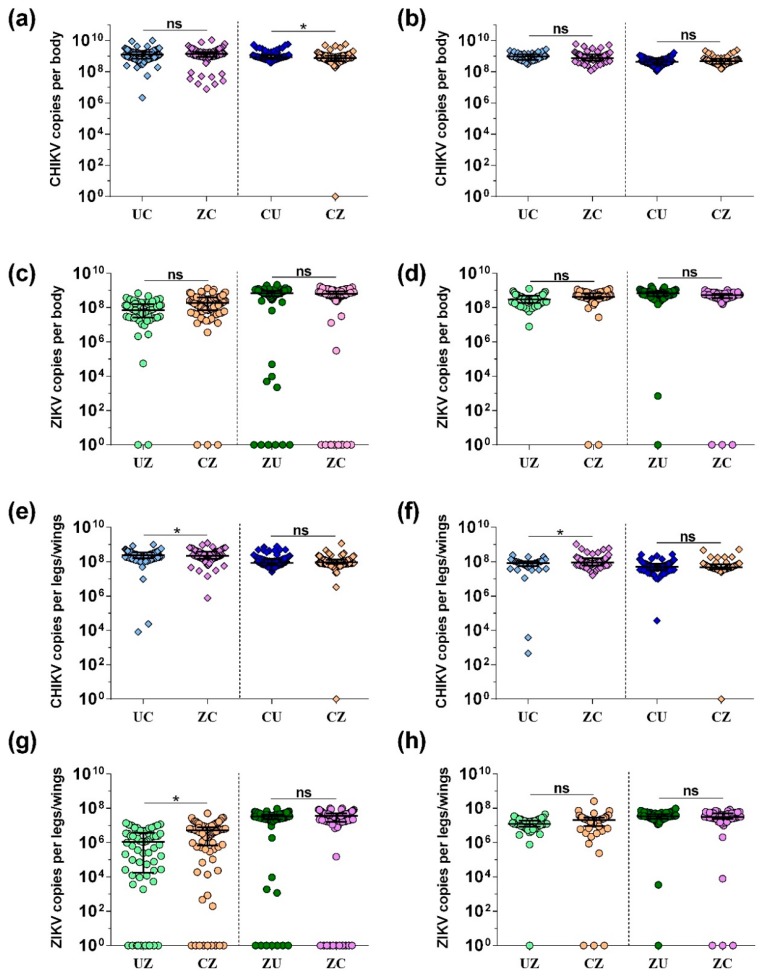
Viral RNA copy numbers in the bodies and legs/wings of single and dual infected *Ae. aegypti* mosquitoes. CHIKV copy numbers in mosquito bodies are shown for (**a**) 7 dpi and (**b**) 12 dpi. ZIKV copy numbers in mosquito bodies are shown for (**c**) 7 dpi and (**d**) 12 dpi. CHIKV copy numbers in mosquito legs and wings are shown for (**e**) 7 dpi and (**f**) 12 dpi. ZIKV copy numbers in mosquito legs and wings are shown for (**g**) 7 dpi and (**h**) 12 dpi. All viral copy numbers were quantified using qRT-PCR and are shown combined from three replicate experiments. The median, including zero values log10(x + 1) transformed, is indicated and error bars show the interquartile range. ANOVA was used to determine statistical significance as indicated (* *p* < 0.05), taking replicates into account.

We next determined whether replication of both viruses in the same mosquito would impact the viral load of either CHIKV or ZIKV in the bodies and legs/wings. We thus compared viral copy numbers (positive-sense viral RNA) in bodies and legs/wings of mosquitoes exposed to only one virus or both viruses sequentially (Figure 2). CHIKV copy numbers were high in the bodies (around 10^9^) and similar between groups. There was no significant difference in CHIKV copy numbers at 7 (Figure 2a) and 12 (Figure 2b) dpi in mosquitoes exposed to CHIKV during their second blood feed (UC vs. ZC). However, there was a small but significant decrease (*p* < 0.05) in CHIKV copy numbers in mosquito bodies exposed to ZIKV after prior CHIKV infection 7 days after the second blood feed (Figure 2a; CU vs. CZ). At 12 dpi, no difference was observed between these groups (Figure 2b; CU vs. CZ). ZIKV copy numbers in mosquito bodies were not significantly different between groups (UZ vs. CZ and ZU vs. ZC) at either 7 (Figure 2c) or 12 (Figure 2d) dpi. Yet, there was a trend for increased ZIKV copy numbers 7 days after the second blood meal with ZIKV if mosquitoes had previously been infected with CHIKV (Figure 2c; CU vs. CZ).

We further quantified how much virus was present within legs/wings (Figure 2e–h). For CHIKV, copy numbers were high (~10^8^). There were marginally significant differences (*p* < 0.05) between CHIKV copy numbers in mosquitoes fed first with ZIKV and then with CHIKV compared to mosquitoes exposed to an uninfectious blood meal first (UC vs. ZC) at both 7 dpi (Figure 2e; 1.05-fold decrease) and 12 dpi (Figure 2f; 1.1-fold increase). There were no differences between mosquitoes fed first with CHIKV and then with an uninfectious or ZIKV-containing blood meal at either time point (Figure 2e,f; CU vs. CZ). ZIKV copy numbers were overall lower (10^6^–10^8^) in legs/wings than CHIKV copy numbers, especially at 7 dpi in mosquitoes that were exposed to ZIKV in the second blood meal (Figure 2g; groups UZ and CZ). These two groups also represent the earliest time point after infection with ZIKV, and copy numbers were increased 5-fold (*p* < 0.05) in mosquitoes that had previously been exposed to CHIKV, compared to the control (UZ vs. CZ). At 12 dpi, there were no significant differences between the groups (UZ vs. CZ and ZU vs. ZC).

### 3.3. Transmission Rates and Viral Load in Saliva

Saliva collection provides a means of estimating the transmission potential of infected mosquitoes. We first determined transmission rates (i.e., proportion of positive saliva samples) in all groups (Table 1, ‘Transmission’). There were no significant differences in CHIKV transmission rates at either 7 or 12 dpi (Table 1; UC vs. ZC and CU vs. CZ). Yet, transmission of CHIKV by mosquitoes first infected with ZIKV was marginally insignificant (*p* = 0.07) (ZC; 67.9% transmitting) compared to mosquitoes first provided an uninfectious blood meal (UC; 42.9% transmitting) 12 days after the second blood meal. In contrast, ZIKV transmission rates significantly increased (*p* < 0.05) at 7 dpi in mosquitoes exposed to both CHIKV and ZIKV compared to single exposure (UZ vs. CZ and ZU vs. ZC), independent of the order of infection. However, there was no difference in transmission rates at 12 dpi for ZIKV (UZ vs. CZ and ZU vs. ZC).

**Figure 3:**
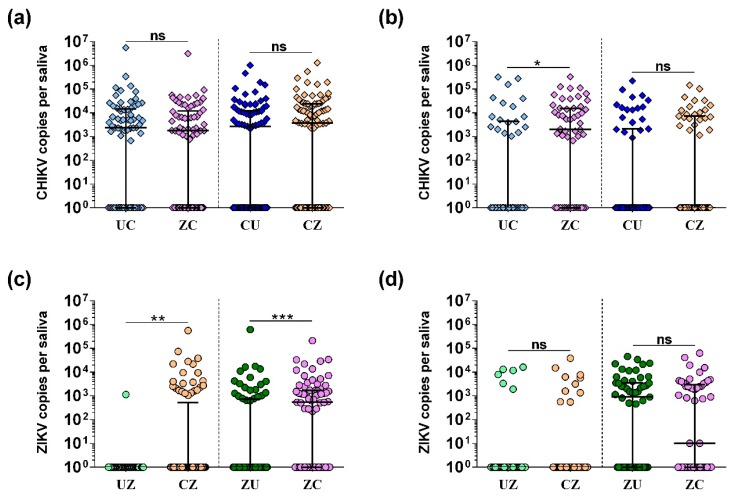
Viral RNA copy numbers in the saliva of single and dual infected *Ae. aegypti* mosquitoes. CHIKV copy numbers in mosquito saliva are shown for (**a**) 7 dpi and (**b**) 12 dpi. ZIKV copy numbers in mosquito saliva are shown for (**c**) 7 dpi and (**d**) 12 dpi. All viral copy numbers were quantified using qRT-PCR and are shown combined from three replicate experiments. The median, including zero values log10(x + 1) transformed, is indicated, and error bars show the interquartile range. ANOVA was used to determine statistical significance as indicated (* *p* < 0.05; ** *p* < 0.01; and *** *p* < 0.001), taking replicates into account.

Next, we quantified viral copy numbers in the saliva (Figure 3). Increased viral load in the saliva may correlate with an increased transmission potential. When comparing viral load in the saliva, we found no significant difference in CHIKV copy numbers at 7 dpi (Figure 3a) between groups (UC vs. ZC and CU vs. CZ). However, we found that, at 12 dpi, CHIKV copy numbers were significantly increased (*p* < 0.05) in mosquitoes that had previously been infected with ZIKV compared to the control (Figure 3b; UC vs. ZC). Additionally, an increase in ZIKV copy numbers was observed 7 days after coinfection compared to the controls both when CHIKV was given first (Figure 3c; UZ vs. CZ; *p* < 0.01) and when ZIKV was given first (Figure 3c; ZU vs. ZC; *p* < 0.0001). ZIKV copy numbers in saliva samples were comparable between single and dual infections at 12 dpi (Figure 3d).

### 3.4. Ae. aegypti Can Co-Transmit CHIKV and ZIKV after Sequential Exposure

It has previously been shown that CHIKV and DENV can be transmitted after simultaneous [19,26,30] and sequential infection [26]. *Ae. aegypti* mosquitoes can also co-transmit CHIKV and ZIKV after exposure with both viruses simultaneously [18,19], but a remaining question was what happens after sequential exposure to CHIKV and ZIKV. Here, we detected both CHIKV and ZIKV in the saliva of 78 mosquitoes by qRT-PCR. At 7 dpi, 20 (22%) and 31 (46%) mosquitoes had double positive saliva in group CZ and group ZC, respectively. At 12 dpi, 6 (12%) and 21 (40%) mosquitoes had double positive saliva in group CZ and group ZC, respectively. We thus suspect that *Ae. aegypti* mosquitoes are capable of transmitting both CHIKV and ZIKV at the same time, following sequential exposure with the two viruses.

## 4. Discussion

Our results show that sequential infection with CHIKV and ZIKV did not lead to a competitive suppression of either virus in mosquitoes but rather slightly enhanced transmission at specific time points. First, the proportion of mosquitoes transmitting ZIKV and the genome copy numbers of ZIKV in saliva was significantly higher at 7 dpi in the groups that received CHIKV prior to or following ZIKV infection. Since viral copy numbers were also enhanced in legs and wings at 7 dpi but transmission rates were comparable by 12 dpi, this data represents a shortened extrinsic incubation period (EIP). The EIP is the time it takes for a mosquito to transmit a virus following an infectious blood meal. EIP is an important factor influencing vectorial capacity, i.e., the overall ability of a mosquito serving as a vector [31]. A reduction in the EIP may have important, non-linear consequences for viral epidemiology. In addition, CHIKV genome copies were significantly higher at 12 dpi when the mosquitoes were infected with ZIKV 7 days prior to CHIKV infection. We also observed a trend that the proportion of mosquitoes with CHIKV-positive saliva (transmission rate) was higher at 12 dpi, corroborating the data on CHIKV genome copies. However, this difference was non-significant (*p* = 0.07), probably due to the low numbers in one out of our three replicates at 12 dpi. The increase in CHIKV copy numbers at 12 dpi in the saliva of mosquitoes that were infected with ZIKV prior to exposure to CHIKV may be considered an extended transmission period. In the control group, which was provided a non-infectious blood meal prior to a CHIKV containing blood meal (group UC), CHIKV-positive saliva dropped off between 7 and 12 dpi. However, in mosquitoes first provided with ZIKV and then with CHIKV (group ZC), saliva positivity was sustained up to 12 dpi. Thus, replication of ZIKV was likely able to prolong transmission in some mosquitoes by an unknown mechanism. Together, these data suggest that sequential exposure to CHIKV and ZIKV has an overall enhancing effect on vector competence. A possible limiting factor in our experiments was the high infection, dissemination, and transmission rates observed, which may have obscured more drastic enhancement effects among the groups.

While identifying the molecular mechanisms leading to this slight enhancement of CHIKV and ZIKV in mosquito saliva are beyond the scope of this work, we can speculate on a few possibilities. Considering that CHIKV infects and replicates relatively quickly in the mosquito ([32], and this study), it is possible that damage of physical barriers caused by CHIKV infection and replication in the mosquito midgut facilitated the dissemination of ZIKV particles that were still in this tissue at the time CHIKV was given. This could explain the higher transmission rates and ZIKV copy numbers in the saliva 7 days after the second blood meal in group ZC as compared to ZU. At 12 dpi, this enhancement was nullified, perhaps because by then ZIKV had also sufficiently disseminated to the salivary glands in the control groups. Although it has recently been shown that infection of *Ae. aegypti* with CHIKV does not increase midgut basal lamina damage caused by a blood meal [33,34], it is possible that CHIKV infection may prolong this damage; however, further studies on damage/disruption of physical barriers in the mosquito by arboviruses are warranted.

Another hypothesis for these enhancing interactions is that the established viruses are overwhelming the mosquito immune system, resulting in less efficient defense against a new incoming virus. CHIKV replicating to high levels will be targeted by the mosquito antiviral RNA interference (RNAi) response [20]. The endonuclease Dicer-2 recognizes and binds dsRNA that is generated as replication intermediates during RNA virus replication and cleaves it into 21nt small dsRNA molecules. One of these strands, the guide strand, is then incorporated into the RNA-induced silencing complex (RISC) with argonaute-2 (Ago-2) at its core. Ago-2 has a so-called slicer activity and will cleave a strand of RNA complementary to the 21nt guide RNA [35]. When CHIKV is replicating to high levels, it may overwhelm this response, allowing ZIKV to replicate in a less controlled manner. Additionally, CHIKV nsP2 and nsP3 have been suggested to antagonize the RNAi response and may thus indirectly enhance ZIKV replication. Similarly, flaviviruses have been shown to antagonize the antiviral RNAi response through subgenomic flavivirus RNA (sfRNA) [36], which may also result in reduced antiviral activity against a second virus infection. Flaviviruses have been shown to induce JAK/STAT and Toll signaling [20], while CHIKV can interfere with mosquito immune signaling pathways, such as the Toll pathway [37]. Thus, if CHIKV or ZIKV are interfering with selected signaling pathways, these viruses may indirectly enhance replication of the coinfecting virus. While there is no strong evidence for such interactions after simultaneous exposure to CHIKV and ZIKV [18,19], we believe that these are dynamic processes potentially requiring established infections or similarly resulting in a fresh boost of replication when a new virus is introduced to an established infection. A possible suppression of antiviral mechanisms by a previous infection could explain the enhancements seen in groups CZ versus UZ (legs/wings and saliva) and ZC versus UC (saliva).

Importantly, if we try to translate our results to field situations, CHIKV or ZIKV arriving in an area where the other virus is already present represents a favorable situation for both viruses and may enhance virus transmission. For instance, in 2015–2016, in an area of the Recife Metropolitan Region, Brazil, we showed that when a ZIKV outbreak was waning, a clear, rapid spread of CHIKV occurred in the region [38]. Although herd immunity was probably the most important cause of the diminishing ZIKV transmission within the human population, we believe that the lack of a negative interaction between CHIKV and ZIKV in the mosquito and a possible enhancement may have been additive factors for the rapid spread of CHIKV. Likewise, if ZIKV is introduced in an area where CHIKV has been circulating, a shortened EIP of ZIKV in mosquitoes previously infected with CHIKV, as seen here, may significantly affect the epidemiology of the newly circulating virus. 

One limitation of our study is that we used only one titer (similar titer of CHIKV and ZIKV) to infect the mosquitoes. It is possible that if the mosquitoes were infected with different titers between the viruses (e.g., higher titer of CHIKV and lower titer of ZIKV or vice-versa), different outcomes might be observed. This is important considering that humans exhibit different viremia levels depending on the arbovirus, e.g., ZIKV viremia is generally lower than CHIKV viremia [10]. Another aspect to be considered is how coinfection (both simultaneous and sequential) may impact virus infection of mosquitoes that have taken only a partial blood meal. In general, mosquitoes fed on artificial membrane feeders are able to fully engorge and are then followed for vector competence. In the field, however, blood meals are more likely to be interrupted, resulting in a partial blood meal. In this scenario, the mosquito will likely feed again after 2–4 days to enable oviposition. Since this would impact both the amount of virus that is ingested, the timing of sequential infection, and the structural changes to the midgut epithelium, it would be interesting to investigate these aspects in the future.

## 5. Conclusions

We have shown here that sequential infection of *Ae. aegypti* mosquitoes with CHIKV and ZIKV did not lead to competitive suppression of either virus and, in fact, enhanced transmission at selected time points. A large number of saliva samples were positive for both CHIKV and ZIKV, providing further evidence that co-transmission of these viruses may occur and could potentially contribute to coinfections of humans in regions where the transmission of these viruses overlaps. Additional studies are needed to further evaluate the role of coinfection in the field, for example, through the testing of individual mosquitoes in endemic areas and how these can affect human coinfections and arbovirus transmission overall.

## Figures and Tables

**Table 1:** Infection (bodies), dissemination (legs/wings), and transmission (saliva) rates for CHIKV and ZIKV after single or sequential dual exposure ^A^.

**CHIKV**	**7 dpi**			**12 dpi**		
	**Infection**	**Dissemination**	**Transmission**	**Infection**	**Dissemination**	**Transmission**
UC	72/72 (100%)	72/72 (100%)	46/72 (63.9%)	42/42 (100%)	42/42 (100%)	18/42 (42.9%)
CU	83/83 (100%)	82/83 (98.8%)	43/83 (51.8%)	59/59 (100%)	59/59 (100%)	19/59 (32.2%)
UZ	-	-	-	-	-	-
ZU	-	-	-	-	-	-
CZ	92/93 (98.9%)	91/93 (97.8%)	55/93 (59.1%)	52/52 (100%)	51/52 (98.1%)	23/52 (44.2%)
ZC	74/74 (100%)	70/74 (94.6%)	45/74 (60.8%)	53/53 (100%)	53/53 (100%)	36/53 (67.9%)
**ZIKV**	**7 dpi**			**12 dpi**		
	**Infection**	**Dissemination**	**Transmission**	**Infection**	**Dissemination**	**Transmission**
UC	-	-	-	-	-	-
CU	-	-	-	-	-	-
UZ	63/65 (96.9%)	55/65 (84.6%)	1/65 (1.54%)	44/44 (100%)	43/44 (97.7%)	9/44 (20.5%)
ZU	74/80 (92.5%)	73/80 (91.3%)	22/80 (27.5%)	54/55 (91.3%)	54/55 (91.3%)	32/55 (58.2%)
CZ	90/93 (96.8%)	83/93 (89.2%)	23/93 (24.7%) ^B^	50/52 (96.2%)	49/52 (94.2%)	10/52 (19.2%)
ZC	63/74 (86.5%)	63/74 (85.1%)	44/74 (59.5%) ^C^	50/53 (94.3%)	50/53 (94.3%)	27/53 (50.9%)

^A^ Combined data from three replicate experiments is shown. Statistical analysis accounted for individual replicates (see Methods). ^B^ ZIKV transmission rates were significantly increased (*p* = 0.0424) in group CZ compared to group UZ at 7 dpi. ^C^ ZIKV transmission rates were significantly increased (*p* = 0.0172) in group ZC compared to group ZU at 7 dpi.

## Supplementary Materials

The following are available online at http://www.mdpi.com/2075-4450/9/4/177/s1, Table S1: Raw data of virus positive tissues from individual replicate experiments, including results of a Chi-Square test to determine heterogeneity among replicates (indicated by *p*-value).
