# Supplementary material for: Sequential Infection of Aedes aegypti Mosquitoes with Chikungunya Virus and Zika Virus Enhances Early Zika Virus Transmission
Source: Insects. 2018 Dec 1;9(4):177. doi: 10.3390/insects9040177 (PMC6315929; doi:10.3390/insects9040177)
Supplement: Supplementary file 1 [file insects-09-00177-s001.pdf]

insects

MDPI

Article

Sequential Infection of *Aedes aegypti* Mosquitoes with Chikungunya Virus and Zika Virus Enhances Early Zika Virus Transmission

Tereza Magalhaes *, Alexis Robison, Michael C. Young, William C. Black IV, Brian D. Foy, Gregory D. Ebel and Claudia Rückert *

Arthropod-Borne and Infectious Diseases Laboratory, Department of Microbiology, Immunology, and Pathology, Colorado State University, Fort Collins, CO 80523, USA; lexir5394@gmail.com (A.R.); emceeyoung@gmail.com (M.C.Y.); William.Black@colostate.edu (W.C.B.I.); Brian.Foy@colostate.edu (B.F.); Gregory.Ebel@colostate.edu (G.D.E.)

* Correspondence: Tereza.Magalhaes@colostate.edu (T.M.); Claudia.Rueckert@Colostate.edu (C.R.); Tel.: +1-970-491-5699 (T.M.); +1-970-491-2202 (C.R.)

Received: 26 October 2018; Accepted: 27 November 2018; Published: date

Supplementary

Table S1. Raw data of virus positive tissues from individual replicate experiments, including results of a Chi-Square test to determine heterogeneity among replicates (indicated by *p*-value).

Replicate	Treatment [1]	Tissue	N infected	N negative	N total	*p*-value	% infected
**7 dpi**							
1	UZ	bodies	22	1	23	1.00	95.7%
2	UZ	bodies	18	0	18		100.0%
3	UZ	bodies	23	1	24		95.8%
1	UZ	legs/wings	19	4	23	0.914	82.6%
2	UZ	legs/wings	15	3	18		83.3%
3	UZ	legs/wings	21	3	24		87.5%
1	UZ	saliva	1	22	23	0.631	4.3%
2	UZ	saliva	0	18	18		0.0%
3	UZ	saliva	0	24	24		0.0%
1	UC	bodies	28	0	28	1.00	100.0%
2	UC	bodies	19	0	19		100.0%
3	UC	bodies	25	0	25		100.0%
1	UC	legs/wings	28	0	28	1.00	100.0%
2	UC	legs/wings	19	0	19		100.0%
3	UC	legs/wings	25	0	25		100.0%
1	UC	saliva	15	13	28	0.116	53.6%
2	UC	saliva	11	8	19		57.9%
3	UC	saliva	20	5	25		80.0%
1	ZU	bodies	25	6	31	**0.00730**[2]	80.6%
2	ZU	bodies	29	0	29		100.0%
3	ZU	bodies	20	0	20		100.0%
1	ZU	legs/wings	25	6	31	**0.0230**[2]	80.6%
2	ZU	legs/wings	29	0	29		100.0%
3	ZU	legs/wings	19	1	20		95.0%
1	ZU	saliva	8	23	31	0.218	25.8%
2	ZU	saliva	11	18	29		37.9%

3	ZU	saliva	3	17	20		15.0%
1	CU	bodies	32	0	32	1.00	100.0%
2	CU	bodies	22	0	22		100.0%
3	CU	bodies	29	0	29		100.0%
1	CU	legs/wings	31	1	32	1.00	96.9%
2	CU	legs/wings	22	0	22		100.0%
3	CU	legs/wings	29	0	29		100.0%
1	CU	saliva	21	11	32	**0.00570**[2]	65.6%
2	CU	saliva	5	17	22		22.7%
3	CU	saliva	17	12	29		58.6%
1	ZC-c	bodies	26	0	26	1.00	100.0%
2	ZC-c	bodies	22	0	22		100.0%
3	ZC-c	bodies	26	0	26		100.0%
1	ZC-c	legs/wings	24	2	26	0.326	92.3%
2	ZC-c	legs/wings	22	0	22		100.0%
3	ZC-c	legs/wings	26	0	26		100.0%
1	ZC-c	saliva	21	5	26	**0.0323**[2]	80.8%
2	ZC-c	saliva	11	11	22		50.0%
3	ZC-c	saliva	13	13	26		50.0%
1	ZC-z	bodies	20	6	26	**0.0410**[2]	76.9%
2	ZC-z	bodies	22	0	22		100.0%
3	ZC-z	bodies	22	4	26		84.6%
1	ZC-z	legs/wings	20	6	26	**0.0428**[2]	76.9%
2	ZC-z	legs/wings	22	0	22		100.0%
3	ZC-z	legs/wings	21	5	26		80.8%
1	ZC-z	saliva	14	12	26	**0.0352**[2]	53.8%
2	ZC-z	saliva	18	4	22		81.8%
3	ZC-z	saliva	12	14	26		46.2%
1	CZ-c	bodies	33	1	34	1.00	97.1%
2	CZ-c	bodies	30	0	30		100.0%
3	CZ-c	bodies	29	0	29		100.0%
1	CZ-c	legs/wings	33	1	34	0.762	97.1%
2	CZ-c	legs/wings	30	0	30		100.0%
3	CZ-c	legs/wings	28	1	29		96.6%
1	CZ-c	saliva	18	16	34	0.604	52.9%
2	CZ-c	saliva	18	12	30		60.0%
3	CZ-c	saliva	19	10	29		65.5%
1	CZ-z	bodies	32	2	34	0.772	94.1%
2	CZ-z	bodies	29	1	30		96.7%
3	CZ-z	bodies	29	0	29		100.0%
1	CZ-z	legs/wings	27	7	34	0.0673	79.4%
2	CZ-z	legs/wings	29	1	30		96.7%
3	CZ-z	legs/wings	27	2	29		93.1%
1	CZ-z	saliva	2	32	34	**<0.0001**[2]	5.9%
2	CZ-z	saliva	5	25	30		16.7%
3	CZ-z	saliva	16	13	29		55.2%
**12 dpi**							
1	UZ	bodies	18	0	18	1.00	100.0%
2	UZ	bodies	16	0	16		100.0%
3	UZ	bodies	10	0	10		100.0%
1	UZ	legs/wings	18	0	18	1.00	100.0%
2	UZ	legs/wings	15	1	16		93.8%
3	UZ	legs/wings	10	0	10		100.0%
1	UZ	saliva	6	12	18	0.306	33.3%

2	UZ	saliva	2	14	16		12.5%
3	UZ	saliva	1	9	10		10.0%
1	UC	bodies	23	0	23	1.00	100.0%
2	UC	bodies	14	0	14		100.0%
3	UC	bodies	5	0	5		100.0%
1	UC	legs/wings	23	0	23	1.00	100.0%
2	UC	legs/wings	14	0	14		100.0%
3	UC	legs/wings	5	0	5		100.0%
1	UC	saliva	11	12	23	0.824	47.8%
2	UC	saliva	5	9	14		35.7%
3	UC	saliva	2	3	5		40.0%
1	ZU	bodies	24	0	24	1.00	100.0%
2	ZU	bodies	27	1	28		96.4%
3	ZU	bodies	3	0	3		100.0%
1	ZU	legs/wings	24	0	24		100.0%
2	ZU	legs/wings	27	1	28		96.4%
3	ZU	legs/wings	3	0	3		100.0%
1	ZU	saliva	12	12	24	**0.00871**[2]	50.0%
2	ZU	saliva	17	11	28		60.7%
3	ZU	saliva	3	0	3		100.0%
1	CU	bodies	23	0	23	1.00	100.0%
2	CU	bodies	22	0	22		100.0%
3	CU	bodies	14	0	14		100.0%
1	CU	legs/wings	23	0	23	1.00	100.0%
2	CU	legs/wings	22	0	22		100.0%
3	CU	legs/wings	14	0	14		100.0%
1	CU	saliva	12	11	23	**0.0385**[2]	52.2%
2	CU	saliva	4	18	22		18.2%
3	CU	saliva	3	11	14		21.4%
1	ZC-c	bodies	25	0	25	1.00	100.0%
2	ZC-c	bodies	21	0	21		100.0%
3	ZC-c	bodies	7	0	7		100.0%
1	ZC-c	legs/wings	25	0	25	1.00	100.0%
2	ZC-c	legs/wings	21	0	21		100.0%
3	ZC-c	legs/wings	7	0	7		100.0%
1	ZC-c	saliva	18	7	25	0.329	72.0%
2	ZC-c	saliva	15	6	21		71.4%
3	ZC-c	saliva	3	4	7		42.9%
1	ZC-z	bodies	22	3	25	0.350	88.0%
2	ZC-z	bodies	21	0	21		100.0%
3	ZC-z	bodies	7	0	7		100.0%
1	ZC-z	legs/wings	22	3	25	0.350	88.0%
2	ZC-z	legs/wings	21	0	21		100.0%
3	ZC-z	legs/wings	7	0	7		100.0%
1	ZC-z	saliva	11	14	25	0.167	44.0%
2	ZC-z	saliva	14	7	21		66.7%
3	ZC-z	saliva	2	5	7		28.6%
1	CZ-c	bodies	27	0	27	1.00	100.0%
2	CZ-c	bodies	20	0	20		100.0%
3	CZ-c	bodies	5	0	5		100.0%
1	CZ-c	legs/wings	27	0	27	0.481	100.0%
2	CZ-c	legs/wings	19	1	20		95.0%
3	CZ-c	legs/wings	5	0	5		100.0%
1	CZ-c	saliva	14	13	27	0.110	51.9%

2	CZ-c	saliva	9	11	20		45.0%
3	CZ-c	saliva	0	5	5		0.0%
1	CZ-z	bodies	26	1	27	0.185	96.3%
2	CZ-z	bodies	20	0	20		100.0%
3	CZ-z	bodies	4	1	5		80.0%
1	CZ-z	legs/wings	26	1	27	0.318	96.3%
2	CZ-z	legs/wings	19	1	20		95.0%
3	CZ-z	legs/wings	4	1	5		80.0%
1	CZ-z	saliva	5	22	27	1.00	18.5%
2	CZ-z	saliva	4	16	20		20.0%
3	CZ-z	saliva	1	4	5		20.0%

[1] For explanation of groups see Figure 1. Added "-c" and "-z" indicate whether reported infection rates refer to CHIKV or ZIKV positivity in these groups, respectively; [2] *p*-values below 0.05 indicate significant variation between replicate experiments for the respective group/tissue. This variation is common in vector competence experiments and not overly concerning. Some variation was found due to a low N at 12 dpi in replicate 3 (low mosquito survival) or lower infection rates following the first blood feed of replicate 1.
